# Quantitatively estimating defects in graphene devices using discharge current analysis method

**DOI:** 10.1038/srep04886

**Published:** 2014-05-08

**Authors:** Ukjin Jung, Young Gon Lee, Chang Goo Kang, Sangchul Lee, Jin Ju Kim, Hyeon June Hwang, Sung Kwan Lim, Moon-Ho Ham, Byoung Hun Lee

**Affiliations:** 1 Center for Emerging Electronic Devices and Systems, School of Materials Science and Engineering, Gwangju Institute of Science and Technology, Oryong-dong 1, Buk-gu, Gwangju, Korea 500-712; 2 Department of Nanobio Materials and Electronics, Gwangju Institute of Science and Technology, Oryong-dong 1, Buk-gu, Gwangju, Korea 500-712

## Abstract

Defects of graphene are the most important concern for the successful applications of graphene since they affect device performance significantly. However, once the graphene is integrated in the device structures, the quality of graphene and surrounding environment could only be assessed using indirect information such as hysteresis, mobility and drive current. Here we develop a discharge current analysis method to measure the quality of graphene integrated in a field effect transistor structure by analyzing the discharge current and examine its validity using various device structures. The density of charging sites affecting the performance of graphene field effect transistor obtained using the discharge current analysis method was on the order of 10^14^/cm^2^, which closely correlates with the intensity ratio of the D to G bands in Raman spectroscopy. The graphene FETs fabricated on poly(ethylene naphthalate) (PEN) are found to have a lower density of charging sites than those on SiO_2_/Si substrate, mainly due to reduced interfacial interaction between the graphene and the PEN. This method can be an indispensable means to improve the stability of devices using a graphene as it provides an accurate and quantitative way to define the quality of graphene after the device fabrication.

The electrical and physical properties of graphene have been extensively investigated for diverse applications such as electronic switch, sensors, transparent electrodes, fuel cells, and catalysts^1^,^2^,^3^,^4^,^5^,^6^,^7^. One of the key common challenges in these diverse applications of graphene is a wide variation in the material quality itself and the influences of the external environment via defects and surface reactions^8^,^9^,^10^,^11^,^12^. Thus, the origins of the instability of graphene devices and their sensitivity to external environmental factors have been extensively investigated^13^,^14^,^15^.

For example, the conductivity of graphene is found to be greatly affected by many factors such as metallic residues originating from the growth processes, substrate materials, capping dielectrics, device fabrication processes such as patterning and cleaning, initial defect density, contact metals, operation ambient, temperature, and more. In addition, the operation history-dependent device drifts such as hysteresis, charge trapping, and interfacial reactions also affect the characteristics of a device using a graphene.

Since the defects in graphene and the influences of the environmental factors play a critical role in device-level operation^16^, methods to quantitatively monitor these factors, especially after device fabrication, are of utmost importance. However, macroscopic defects in graphene substrates have been primarily characterized using physical analysis methods such as visual inspections, Raman spectroscopy, atomic force microscopy (AFM), transmission electron microscopy (TEM), and scanning tunneling microscopy (STM)^17^,^18^,^19^. The distance between defects, grain size, and relative density of sp^3^ type defects can be measured using the shift in the relative position and width of the G-peak, D-peak, and 2D-peak of the Raman spectrum. Various TEM and STM analyses of grain size, defects, and contaminants have been reported in the literature^19^,^20^.

While Raman spectroscopy, AFM, TEM, and STM are useful means in the analysis of the initial quality of graphene, these methods are not easy to use when examining graphene integrated in a device structure^21^,^22^,^23^. For example, Raman spectroscopy becomes inaccurate when the graphene has a dielectric passivation layer. The beam size of a Raman spectrometer, which is typically larger than 0.5 μm, is too big for applications requiring small graphene patterns. Furthermore, Raman analysis cannot be performed with a thick metal electrode on graphene. AFM, TEM, and STM have similar limitations as they are destructive analysis methods. Thus, it has not been possible to quantitatively analyze the quality of graphene at the device level, which is critically important for practical applications.

Thus, the characteristics of graphene integrated in a device structure have been primarily investigated indirectly using the electrical characteristics of devices such as current-voltage (I-V) or capacitance-voltage (C-V) characteristics^22^,^23^,^24^, which provide information on the mobility, location of the Dirac point, and hysteresis of the I-V curves^26^. Unfortunately, these device characteristics are known to vary significantly because they are very sensitive to environmental factors (substrate, capping dielectric, ambient, temperature, contact resistance, etc.)^27^,^28^. Since these aforementioned methods do not provide a direct means of analyzing the quality of graphene after device fabrication, it is imperative to develop a new method to quantitatively identify the influence of defects and environmental factors even after device fabrication. Without a direct defect assessment method, it will be impossible to identify whether the changes in the device characteristics are induced by changes in the quality of the graphene or by the influence of extrinsic factors.

In this work, a new electrical characterization method to obtain quantitative information on defects and other environmental factors representing the quality of graphene FETs has been proposed and its validity has been examined using devices fabricated on a large area monolayer graphene sheets with different levels of physical defect density (see Methods). This method, called the discharge current analysis (DCA) method, modulates the carrier concentration of graphene periodically using an external pulse bias and translates the frequency dependence of the charges discharged from the graphene channel into a density of charging sites. The validity of this DCA method is examined by correlating the electrically measured density of charging sites with the initial physical defect density of graphene measured with Raman spectroscopy.

For silicon MOSFETs, a charge pumping (CP) method has been used to accurately analyze the interfacial defect density, which affects the device performance. The CP method fills up defect sites at the silicon-dielectric interface using minority carriers supplied from the source and drain at an inversion state, as shown in Fig. 1a. Then, majority carriers from the substrate are supplied to the interface by changing the silicon surface to an accumulation state. The majority carriers are supplied to the defects only in the channel because they cannot flow into the source/drain side due to the energy barrier of the pn junction. Finally, the defect density can be calculated by counting the number of majority carriers recombined with the minority carriers in the defect sites. The measurement principle of CP is explained in detail in the Supplemental Section S1. Unfortunately, this method cannot be used for graphene FETs because there is no body contact to supply the majority carriers to the channel. As a detour, the carriers charged and released from defect sites near the surface of graphene or graphene itself can be collected through the source/drain as shown in Fig. 1b. This approach is feasible for a graphene because there is no barrier for the carriers due to Klein tunneling. However, the separation of carriers from the defect sites and the channel itself becomes a technical challenge because majority carriers in the graphene channel and nearby defect sites are collected together. If the carriers from defect sites can be separated from the total discharge current measured at the source/drain, the status of graphene and its environment can be systematically investigated.

Figure 1c schematically shows the charge supply and discharge process in a graphene channel during a single pulse application. At the onset of a negatively biased gate pulse, the potential of the graphene decreases and hole population in the channel increases (step 2). This is equal to moving to a higher current point in the current-voltage curve of a graphene MOSFET, as shown in Fig. 1d. The speed of response at step 2 is comparable to the dielectric relaxation time around ps. Then, defect sites in the graphene and the nearby interfaces are filled up (step 3). The speed of response at step 2 varies depending on the types of defect sites and on the order of μs to ms. When the gate pulse is off, the overly populated carriers in the channel are discharged through the source and drain contact within a dielectric relaxation time (step 4). However, the charges trapped in various trap sites are slowly discharged with a time constant in a range of μs to a few seconds (step 5). As a result, the tail portion of the discharge current, *δI*, highlighted in red in Fig. 1c, contains the charges released from the defect sites of the graphene as well as other surface states near the graphene (to be called charging sites), which discharges more slowly than the discharge from the bulk of the graphene.

The baseline current, *I_o_*, does not change as a function of frequency because it is proportional to the total width of the pulse peak as shown in Fig. 2a. However, the additional current, *δI*, discharged from the charging sites in each cycle increases as a function of frequency. Thus, the discharge current can be approximately represented using the following equation, 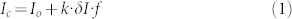
where *I_c_* is the measured discharge current, *I_o_* is the current due to the charges accumulated in the graphene itself, *k* is a frequency-dependent loss factor to account for the charge loss to the source side due to asymmetric metal contacts and other factors, *δI* is the additional discharge current from charging sites, and *f* is the measurement frequency.

Typical discharge current measured at 10 KHz to 1 MHz from CVD-grown graphene FETs is shown in Fig. 2b. In this work, we used large-area monolayer graphene and a number of devices having variations in the level of physical defect density were fabricated at the same time (see Methods and Supplementary Fig. S2). The discharge current, *I_c_*, is linearly proportional to the frequency in the 10 KHz to 100 KHz range. As mentioned above, the tail current, *δI*, consists of charges released from defects sites or other surface states. The slope slightly increases above 100 KHz and then saturates at a higher frequency.

To understand the frequency response of the *I_c_* curve, the charge trapping and detrapping mechanism in the graphene should be explained first. Recently, our group suggested that the hysteresis of a graphene I-V curve is primarily caused by two representative mechanisms, tunneling and a surface redox reaction^31^. The tunneling-induced charge trapping occurs within a few tens of a μs time constant. The time constant of the surface redox reaction is on the order of a few hundred μs to ms. Here, the definition of time constant is the time to generate 63% of charging. Since the time constant of the tunneling process is on the order of 10 μs, the tunneling-induced discharge current can be modulated up to a few hundred KHz, but the discharge current originating from the surface redox reaction cannot follow the gate modulation even at a few tens of KHz. Thus, the contribution from the surface redox reaction is negligible from 10 to 100 KHz. However, this limit does not impede measuring defect density because the amount of the surface redox reaction is relatively small compared to the tunneling, and it is very weakly related to the initial defect density measured using the I(D)/I(G) ratio^16^,^30^.

In the 10 KHz to 100 KHz range, *I_c_* primarily monitors the discharge current due to the tunneling component, and it is proportional to the initial defect density as shown below. At this frequency range, *I_c_* linearly increases in proportion to the frequency. Thus, the slope of the linear portion can be used to extract the density of charging sites. On the other hand, at a frequency above 100 KHz, charge traps that cannot release charges within 5 μs start to continue holding the charges and those traps no longer contribute to *I_c_*. As a result, the slope of *I_c_* increases and eventually becomes saturated as shown in steps 5 and 6 of Fig. 2b. Data obtained from above the saturation limit should be ignored because the gate pulse cannot be applied correctly to the graphene channel at this frequency due to the high channel impedance of the long channel graphene FETs used in this work.

Figure 2c shows I_c_-f curves for four representative devices with different initial defect densities. All four curves show two regions of different slopes as a function of frequency at 20 to 100 KHz and 100 to 150 KHz, respectively. The *I_c_* curves are saturated at ~200 KHz and above. The linear slope at 20 to 100 KHz is attributed to the charge exchange between the charge sites and the graphene through the tunneling mechanism explained above. To investigate the influence of initial defect density (defined as I(D)/I(G) ratio) of graphene, we conducted Raman spectroscopy on each graphene channel after the graphene channel patterning (Supplementary Fig. S2), and then measured the electrical properties after the completion of device fabrication. Interestingly, *δI* value of thirty five devices are found to be closely correlated with the initial defect density, I(D)/I(G) as shown in Fig. 2d. This correlation indicates that the *δI* value representing the density of charging sites originating from both the graphene bulk channel and various charging sites near the graphene can be used to extract the density of graphene defect sites even after device fabrication.

On the other hand, the second slope at 100 KHz to 150 KHz is attributed to the change in the base current, *I_o_*, due to the Dirac point shift during the measurement. Steps 5 and 6 of Figure 2a show that the effective charge density at the pulse peak is maintained even during the pulse off cycle due to the slow discharge. This is equivalent to the parallel shift of I-V curves as shown by the blue curve in Fig. 1d. This gradual increase in the charge density in the channel region shifts the Dirac point to the right side and increases *I_o_*. The second slope showed a strong temperature dependence, indicating that it is related to the thermally activated mechanism such as chemical reaction.(Supplementary Fig. S4) Thus, the second slope should be used to monitor the differences in the slow discharge components rather than defect monitoring, which represents environmental factors such as water molecules trapped around the graphene.

The slope, *δI/δf*, can be translated into density of charging sites using following equation, 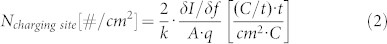
where *A* is the device area and *N_charging site_* is the density of charging sites that incurs charging trapping. The value is multiplied by a factor of 2 because nearly half the discharge current goes to the source side due to a very small drain bias and a symmetrical band structure. Also, some of the charges can be dissipated by recombination at the charging sites. Even though it is not easy to assess the exact amount of charge loss, the ratio of charge loss can be simply represented using a constant charge loss factor, *k*. A typical *k* factor was 0.6 – 0.7 in 20 to 100 KHz region for the devices used in this work.

Finally, *δI/δf* values are converted to the density of charging sites, i.e., *N_charging site_*, and correlated with the initial defect density, I(D)/I(G) (Fig. 2d). *N_charging site_* values extracted in this work are on the order of 10^14^/cm^2^. This value is relatively high compared to the typical interface state density of silicon MOSFETs, which are on the order of 10^10^/cm^2^ to 10^13^/cm^2^ 33,34. However, considering the initial quality of CVD graphene is not as good as exfoliated graphene and the charging sites include all the defect sites in the graphene channel and other trap states near the graphene, this value does not seem unreasonable^35^.

There is appreciable data scattering in Fig. 2d. A part of the data scattering is due to the limited accuracy of Raman analysis, which represents the quality of graphene only within the beam diameter of ~1 μm while the charging current is collected from the whole effective active channel area. Also, the scattering in the charging current measurements, which is due to the influence of hysteretic device characteristics, should be accounted for. Despite these uncertainties, the density of the charging sites correlates remarkably well with the initial defect density, indicating that the I_c_-f measurement method can be instrumental in analyzing graphene quality, especially after device fabrication, because this method can be used to compare the impact of processes on the same graphene even without calibration.

The DCA method may look similar to the charge pumping method, which measures the interface state density of silicon MOSFETs, but the measurement principle is quite different^31^,^32^. In the DCA method, the slope of the discharge current-frequency (I_c_-f) curve is used to analyze the density of the charging sites while the electron-hole pair recombination at the interfacial charging sites is measured at a fixed frequency in the charge pumping method.

Figure 3 shows examples of various graphene FETs structures analyzed using the DCA method. Figure 3a shows the I_c_-f curves measured in bottom gate graphene FETs with/without a 30 nm Al_2_O_3_ passivation layer. Since the Al_2_O_3_ passivation decreases contamination from the graphene surface, the density of the charging sites decreased to 2.47 × 10^14^/cm^3^ from 3.19 × 10^14^/cm^3^ after passivation. A more prominent slope increase before the passivation in the 100 to 180 KHz region indicates that the graphene surface is more prone to the water redox-induced charge generation, as expected^25^,^36^,^37^,^38^. A second example compares the top gate graphene FETs on a 90 nm SiO_2_/silicon substrate with a poly(ethylene naphthalate) (PEN) substrate (Fig. 3b). In this case, 30 nm Al_2_O_3_ is used as the gate dielectric layer for both device structures. Since the PEN substrate is hydrophobic, the *I_o_* shift due to the water redox reaction is expected to be much less pronounced than that of the SiO_2_ substrate^8^. Also, more stable I-V characteristics have been reported for hydrophobic substrates^39^,^40^,^41^. Thus, the density of charging sites should be lower with graphene on PEN substrates. Even though the initial quality of the graphene was similar, the density of charging sites in the graphene FET on PEN ~6.25 × 10^13^/cm^2^ was much less than ~6.66 × 10^14^/cm^2^ of the SiO_2_/Si substrate. Even though the charge loss factor, *k*, was very low ~0.2 for top gate graphene FETs because of fast recombination in the charging sites at the Al_2_O_3_ interface, a relative comparison between different substrates was feasible. The much lower *N_charging site_* in the PEN substrate is primarily attributed to the reduced interaction between the graphene and PEN substrate. These examples clearly demonstrate that the new DCA method can be used to assess the influence of environmental factors and the quality of graphene after device fabrication.

Even though this unique electrical characterization method is the first attempt to quantitatively analyze the density of charging sites of graphene FETs after device fabrication, the preceding examples show how instrumental this method can be in optimizing graphene device structures. Similar to the concept of charge pumping for silicon MOSFETs, which has evolved into several advanced CP methods, there might be several modifications that can be made to improve the accuracy of this method and enhance the quality of information extracted from graphene FETs. Yet, the DCA method proposed in this work will provide an important stepping stone towards the realization of high quality graphene FETs.

## Methods

### Methods summary

Experimental graphene samples were prepared through transfer process and conventional photolithographic technique using graphene sheets grown on copper foil by a CVD method. Before the completion of the device fabrication, Raman spectroscopy was performed on the patterned graphene channel. Electrical properties of fabricated graphene FETs are examined with continuous pulse system. In order to monitor the defect density of graphene in a complete device structure, discharge current was measured by applying square pulse biases at 10–1,000 KHz using a pulse generator and a parameter analyzer.

### Graphene synthesis and transfer

A 1 cm × 1 cm monolayer of graphene sheet grown on Cu foil using a chemical vapor deposition (CVD) process was transferred to 90 nm SiO_2_ thermally grown on P-doped Si substrate using a PMMA-mediated transfer method^29^,^30^. The optical photograph and the Raman data of graphene used in this work showed that the graphene is mostly a monolayer with the low defect density (Supplementary Fig. S2).

### Fabrication of graphene FET devices

For the device fabrication, 100 nm Au electrode patterns were formed on a graphene sheet using i-line contact photolithography and a lift off process. Then, graphene channels between source and drain electrode were patterned using i-line photolithography and oxygen plasma ashing process (process power = 50 W, process time = 90 seconds). The surface of graphene channel was passivated with 30 nm Al_2_O_3_ using atomic layer deposition (ALD) process at 130°C and annealed in N_2_ ambient at 200°C for 30 min to minimize the influence of ambient.

### Raman spectroscopy and mapping

After the graphene channel patterning, the quality of graphene channel defined as I(D)/I(G) ratio was measured for all devices using Raman spectroscopy (Renishaw, λ = 248 nm, power = 20 mW). Supplementary Fig. S2b shows the representative Raman spectra of the single-layer CVD graphene. To investigate the influence of the initial defect density, 35 graphene channels with various I(D)/I(G) ratio from 0.13 to 0.41 are chosen from various locations of graphene sheet as shown in Supplementary Fig. S2c and S2d. Electrical characteristics of these devices are monitored after the completion of device fabrication. (Supplementary Fig. S3).

### Discharge current measurements

**Figure 1:**
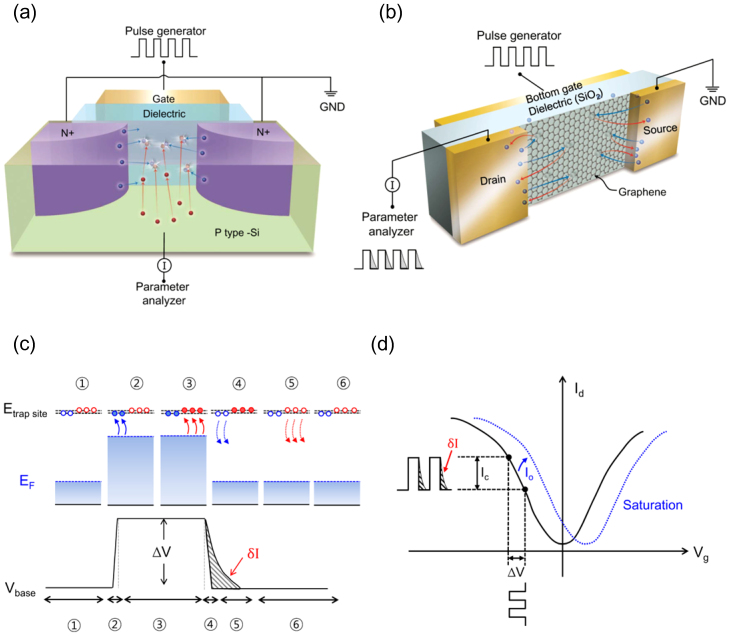
A discharge current analysis method for graphene devices. (a), (b), Schematic showing the electrical connection and charge flow direction to measure the interfacial defect density for silicon MOSFETs (a) and the charging site density for graphene FET(b). (c), Schematic showing the sequence of discharge current generation in a single pulse cycle. Blue circle represents the intrinsic bulk carriers and red circle represents diverse defect sites, which has much slower charging and discharging time than the intrinsic bulk carriers. Step 1 is the initial state before applying a pulse. Holes are populated by the pulse (Step 2), Then, defect sites are charged up in step 3. When the pulse is turned off, majority carriers are discharged quickly(Step 4), and then trapped charge are slowly discharged(Step 5). Finally, channel charge density returns to the initial state. (d), I-V curve of a graphene FET showing the range of pulse bias. The discharge current is generated by a continuous pulse with a pulse height of ΔV.

For discharge current analysis, square pulse biases in 10 KHz to 500 KHz range were applied to the substrate, which works as a bottom gate electrode with 90 nm SiO_2_ gate dielectric, using a pulse generator (Agilent 81110) as shown in Fig. 1a. Pulse width was determined by the duty cycle of pulse ( = 50%) at a given frequency and the rise and fall time was fixed as 100 ns. The source is connected to a ground and the drain is connected to a parameter analyzer (Keithley-4200 SCS) for a current measurement.

## Author Contributions

**Figure 2:**
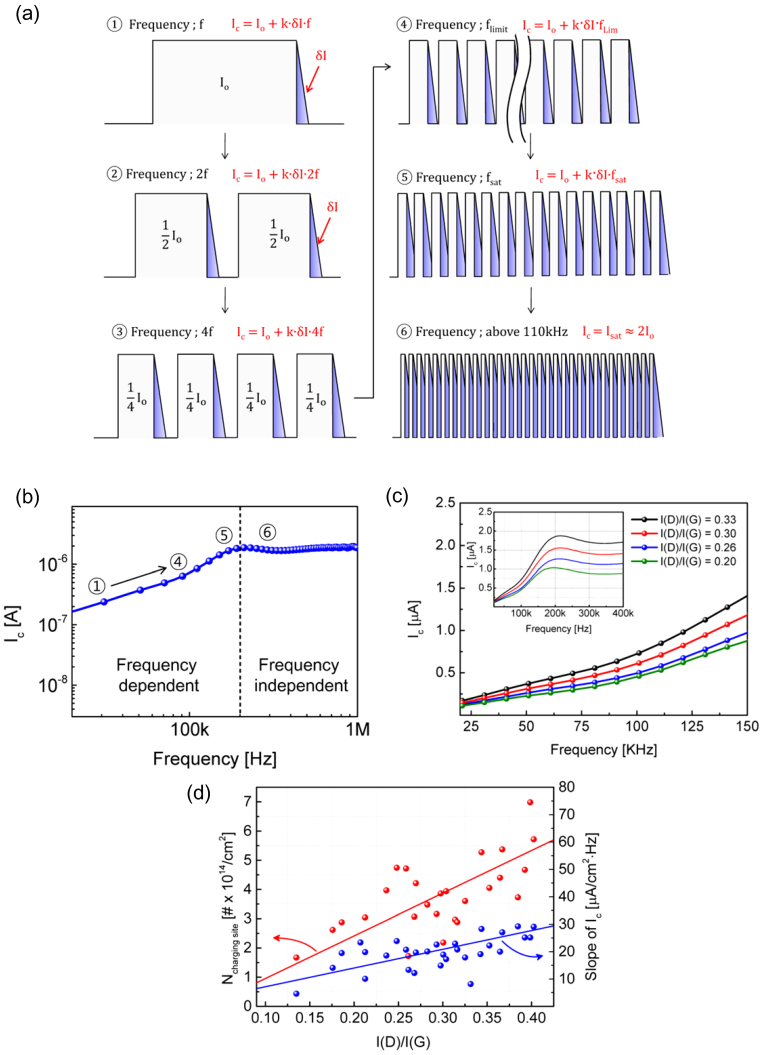
Frequency dependence of discharge current from graphene FETs. (a), Schematic pulse trains showing the amount of discharge current as a function of measurement frequency. Step 1 shows total discharge current(I_c_) including base current (I_o_) and discharge current (δI) at frequency f. As shown in step 1 to step 4, δI increases as the frequency increases. Step 4 shows the boundary case where the pulse off time is equal to the discharge time. Then, the effective pulse on cycle increases effectively and the slow charging mechanism is activated as shown in step 5. Finally, I_c_ is saturated by the impedance limit and slow discharge mechanism(step 6). (b), Representative frequency dependence of the discharge current (I_c_-f) of a graphene FET. (c), I_c_-f measurements for four representative graphene FETs with different initial defect densities. Inset shows the full range of frequency dependence. (d), The slope of the I_c_ at a frequency lower than 100 kHz (right axis) and the density of charging sites (*N_charging site_*) (left axis) are correlated with the initial defect density, I(D)/I(G).

**Figure 3:**
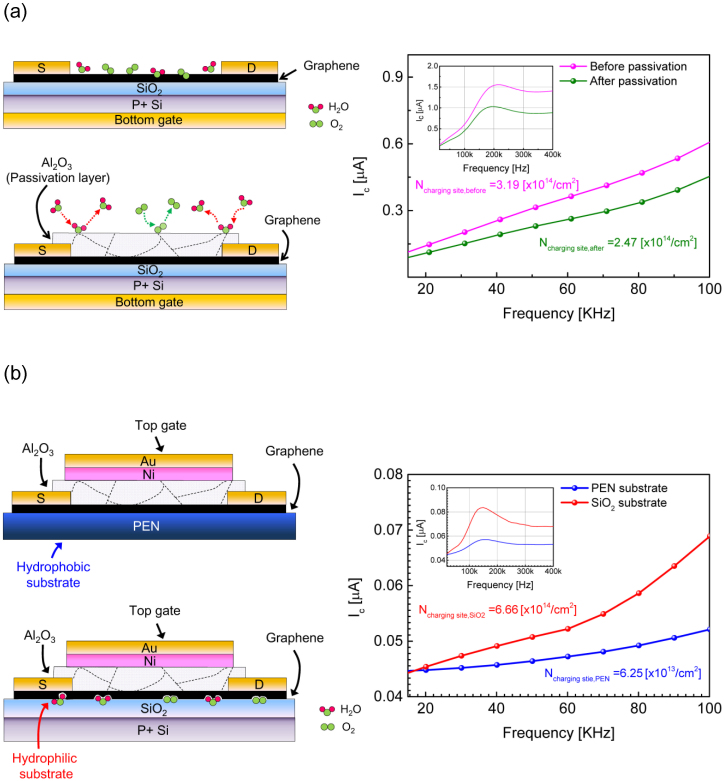
Application of various graphene FETs structures. (a), Device structure (left) and I_c_-f curves (right) of bottom-gated graphene FETs with and without a 30 nm Al_2_O_3_ passivation layer. The passivation layer blocks the interaction of graphene with oxygen and water-related adsorbates. (b), Device structure (left) and I_c_-f curves (right) of top-gated graphene FETs on poly(ethylene naphthalate) (PEN) substrates and 90 nm-thick SiO_2_/silicon substrates. The 30 nm-thick Al_2_O_3_ layer was used as a top dielectric. The graphene on PEN showed much lower defect density than the graphene on SiO_2_/Si.

U.J. performed electrical measurement and data analysis and worth the main manuscript with M.-H.H. and B.H.L., J.J.K. and H.J.H. performed the electrical measurement of devices and data analysis. Y.G.L. programed lab view software to collect the data presented in Fig. 2 and Fig. 3. C.G.K. and S.L. fabricated graphene FET on oxide and PEN substrate used in Fig. 3 respectively. S.K.L. developed the graphene process and fabricated the graphene sheet used in this work. M.-H.H. and B.H.L. wrote the main manuscript and jointly developed a model for data analysis. All authors reviewed the manuscript.

## Supplementary Material

Supplementary Information Supplemetal data
