# Supplementary material for: Quantitatively estimating defects in graphene devices using discharge current analysis method
Source: Sci Rep. 2014 May 8;4:4886. doi: 10.1038/srep04886 (PMC4013935; doi:10.1038/srep04886)
Supplement: Supplementary Information — Supplemetal data [file srep04886-s1.pdf]

Supplementary information

Quantitatively estimating defects in graphene devices using discharge current analysis method

[1]Ukjin Jung, [1]Young Gon Lee, [1]Chang Goo Kang, [2]Sangchul Lee, [2]Jin Ju Kim, [1]Hyeon June Hwang, [2]Sung Kwan Lim, [1,2]Moon-Ho Ham, and [1,2]Byoung Hun Lee*

[1]Center for Emerging Electronic Devices and Systems, School of Materials Science and Engineering, Gwangju Institute of Science and Technology, Oryong-dong 1, Buk-gu, Gwangju, Korea 500-712

[2]Department of Nanobio Materials and Electronics, Gwangju Institute of Science and Technology, Oryong-dong 1, Buk-gu, Gwangju, Korea 500-712

Corresponding author: Byoung Hun Lee (bhl@gist.ac.kr)

Table of contents

Charge pumping technique in MOSFETs (Figures S1) **S3**

Characterizations of graphene (Figures S2) **S5**

DC-IV characteristics of a graphene MOSFETs (Figure S3) **S6**

Temperature dependence of the discharging current measurement (Figure S4) **S7**

References **S8**

Charge pumping technique in MOSFETs

Charge pumping method is well known method used to quantitatively measure the semiconductor-dielectric interface trap density of MOSFETs [2]. This technique measures the recombination current consists of majority carriers from a silicon substrate in the weak inversion state.

To measure charge pumping current, the source and drain of MOSFETs are tied together. The voltage pulse is applied to the gate using a pulse generator. The peak and bottom voltage of pulses applied to a gate is set to modulate the surface potential of silicon substrate from an accumulation to an inversion state. Minority carriers supplied from drain and source fill up the channel and defects states during an inversion state as shown in Figure S1(a). Then, during the accumulation cycle, majority carriers in the substrate, i.e. holes in this example, recombines with the electrons trapped at the defect sites as shown in Figure S1(b). The holes annihilated through electron-hole pair recombination process is replenished from the substrate side. Then, the charge pumping current to supply the holes to the surface of silicon substrate is measured from back gate contact.

Interfacial defect density, $N_{it,}$ can be calculated from the charge pumping current, I_{cp}, using following the equation.

$$N_{it} = I_{cp} / (q \cdot f \cdot A)$$

where, I_{cp} is charge pumping current, f is test frequency, q is electron charge (1.6 x 10^{19}C), and A is channel area in cm^2.

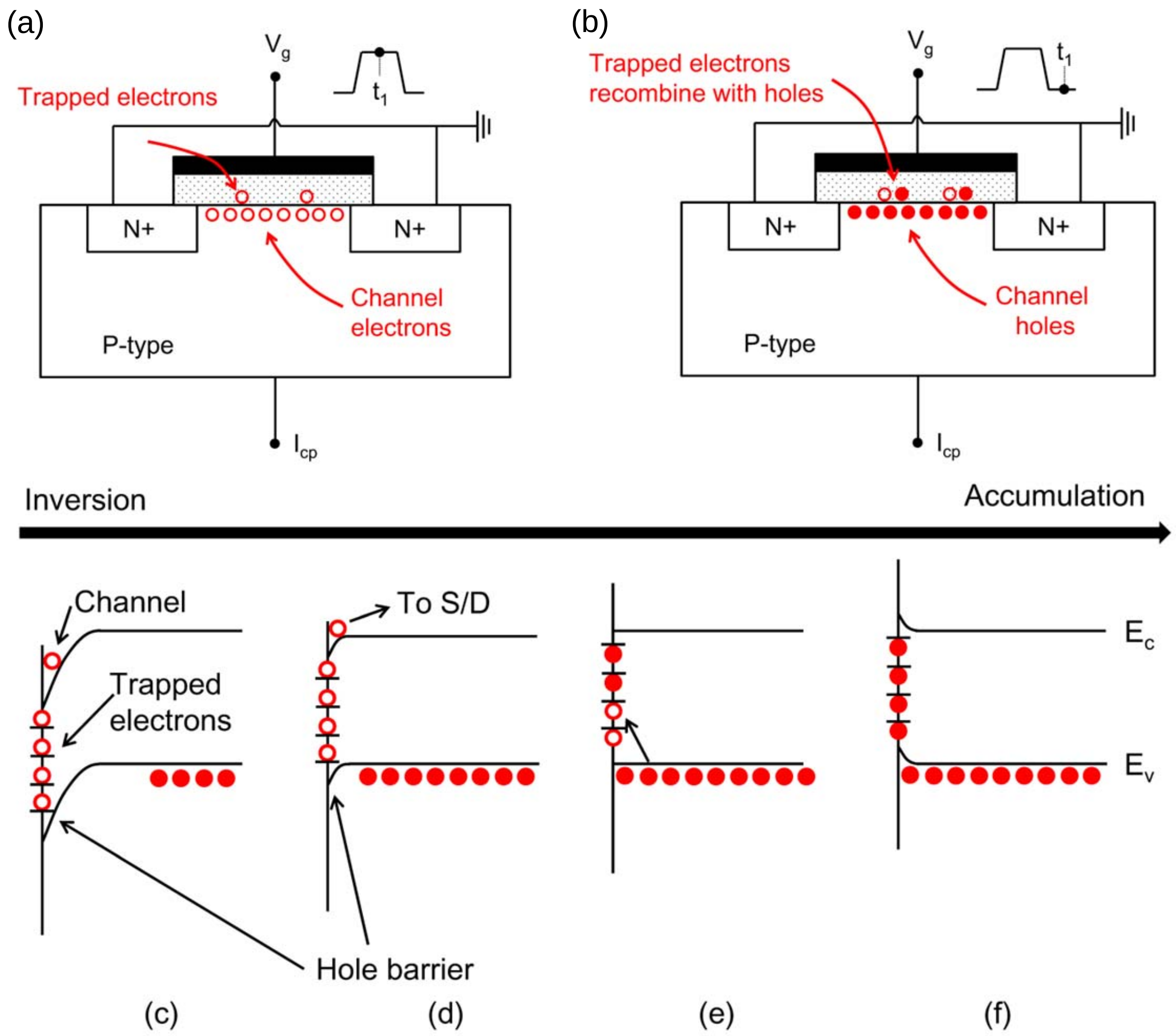

Figure S1. Theory of silicon charge pumping technique (a) Schematic of MOSFETs in an inversion state. (b) Schematic of MOSFETs in an accumulation state. (c)–(f) shows the band diagrams showing the electron trapping and hole recombination process during one cycle of gate pulse.

Characterizations of graphene

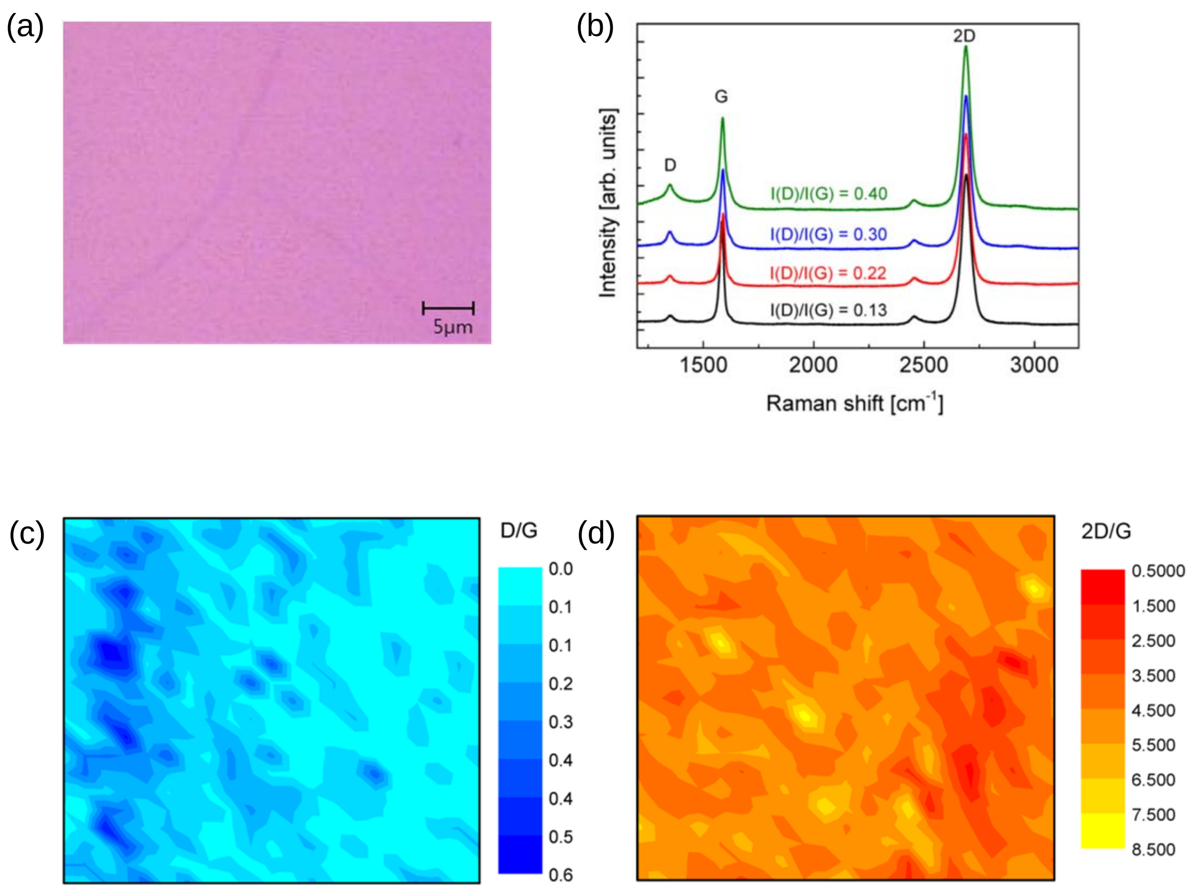

Figure S2. Optical image and Raman spectra of graphene used in this work a. optical image of single layer CVD graphene sheet. **b.** Representative Raman spectra of four graphene FETs having different initial defect density values. **c.** The mapping image of the D-peak/G-peak areal ratios, which are proportional to the physical defect density of graphene [17]. Mapping images show 21 x 21 spots of 10 μm x 10 μm devices **d.** The mapping image of the 2D-peak/G-peak areal ratios showing the thickness uniformity of graphene.

DC-IV characteristics of a graphene MOSFETs

Fig. S3a shows DC I_d-V_g curves as a function of channel length. Even though there is considerable scattering in the drain current, the drain current per unit channel width is similar at wide range of channel width.

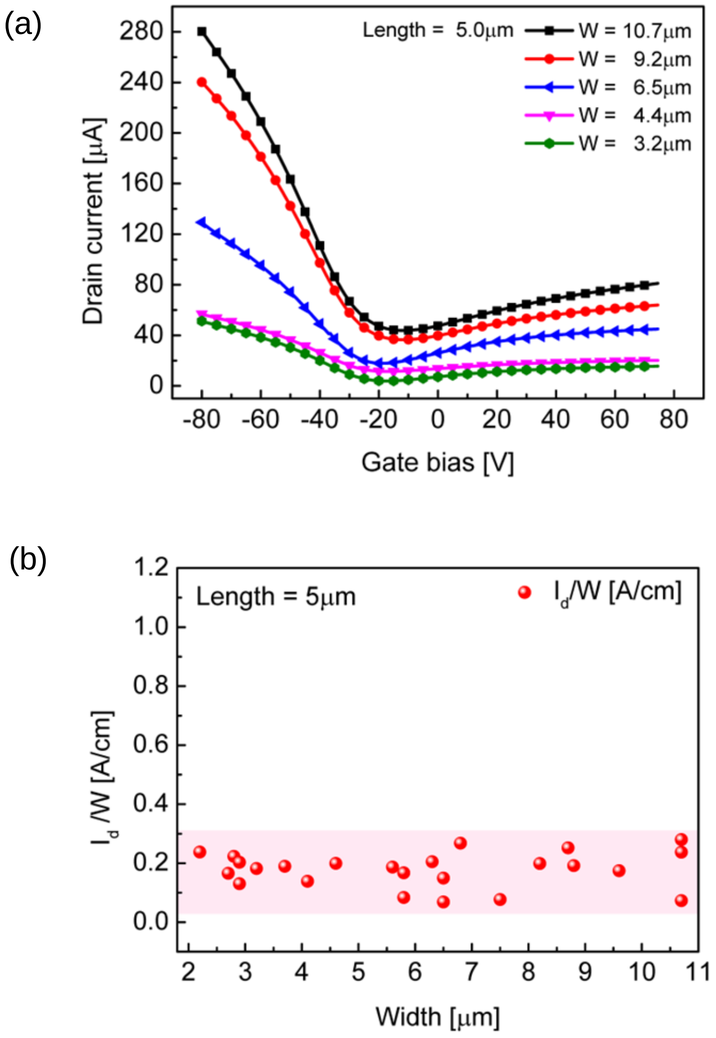

Figure S3. Electrical characteristics of graphene MOSFETs; The drain current (I_d) versus gate bias (V_g) with a fixed drain bias (V_d) of 100 mV. (a) Representative I_d-V_g characteristics (b) Drain current properties per unit width at channel widths from 2 μm to 10.7 μm at fixed channel length = 5 μm.

Temperature dependence of the discharging current measurement

The slope of I_c increases at higher temperatures, especially at a frequency range from 100 KHz to 200 KHz. This change is due to the increased chemical reaction at the surface of the graphene[1]. The slope starts to increase at a lower frequency as the temperature increases because the chemical reaction dominates the discharge current at high temperatures.

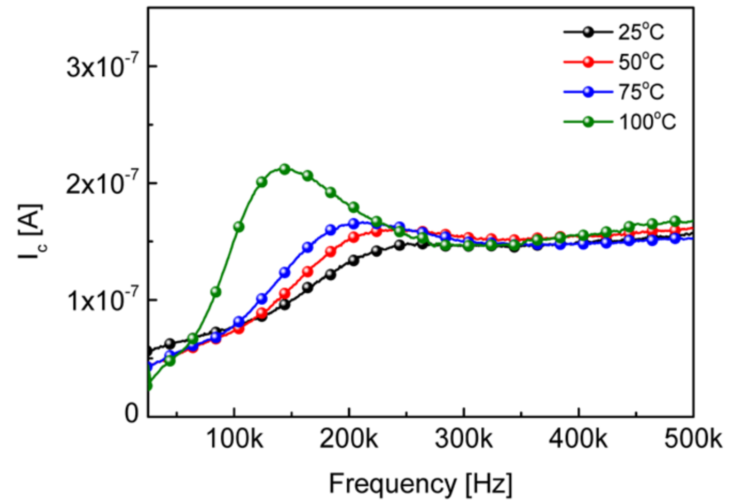

Figure S4. Temperature dependence of the discharging current measured at temperatures from 25 °C to 100 °C.

Reference

1. Chen, J.-H., Jang, C., Xiao, S., Ishigami, M. & Fuhrer, M. S. Intrinsic and extrinsic performance limits of graphene devices on SiO_2. *Nat. Nanotechnol.* **3,** 206–209 (2008).
2. Groeseneken, G., Maes, H. E., Beltran, N. & De Keersmaecker, R. F. Reliable Approach to Charge Pumping Measurement in MOS transistors. IEEE *Trans. Electron Devices* **ED-31,** 42–53 (1984).
